# Oral Adverse Drug Reactions in Patients Receiving Polypharmacy: A Prospective Interdisciplinary Case Series

**DOI:** 10.7759/cureus.117405

**Published:** 2026-10-03

**Authors:** Jessica Singh, Neena Bhatti, Girish Joseph, Jatinder Singh, Manhardeep Kaur, Dharminder Ahir

**Affiliations:** 1 Dentistry, Gian Sagar Dental College and Hospital, Rajpura, IND; 2 Pharmacology and Therapeutics, Christian Medical College and Hospital, Ludhiana, IND; 3 Pharmacology, National Institute of Medical Sciences (NIMS) University, Jaipur, IND; 4 Community Medicine, Government Medical College, Patiala, IND

**Keywords:** adverse drug reaction, gingival overgrowth, oral medicine, pharmacovigilance, polypharmacy, xerostomia

## Abstract

Background and objective: Polypharmacy in older adults markedly increases the risk of adverse drug reactions (ADRs). While the systemic pharmacokinetic and pharmacodynamic consequences of polypharmacy are extensively documented, the oral cavity remains an under-evaluated site for both mucosal and functional drug toxicity. Drug-induced oral manifestations range from dysgeusia and xerostomia to gingival overgrowth, lichenoid reactions, erythema multiforme, opportunistic infection, angioedema, and medication-related osteonecrosis of the jaw, and they meaningfully impair oral health-related quality of life. This case series was undertaken to characterize oral ADRs in patients on polypharmacy through a targeted, interdisciplinary pharmacovigilance approach. This study aims to identify and characterize the spectrum of oral ADRs in patients exposed to polypharmacy, determine the drug classes most frequently implicated, and assess the causality of each reaction using a standardized probability scale.

Methods: This prospective, observational case series was conducted over six months at a tertiary care teaching hospital. Ten adult patients taking five or more concurrent systemic medications who developed new-onset oral lesions or functional oral impairment were enrolled after written informed consent. The evaluation was delivered through an interdisciplinary collaboration led by a dentist, together with the Departments of Pharmacology and Community Medicine. Comprehensive medication reconciliation and a structured oral examination were performed, and causality was assessed using the Naranjo Adverse Drug Reaction Probability Scale.

Results: Ten patients (mean age 67.8 years; 60% male) taking between five and eight concurrent medications were evaluated. The most frequent oral ADRs were functional salivary impairment (xerostomia, 20%) and structural modification (gingival overgrowth, 20%). The agents most often implicated were antihypertensives (calcium channel blockers and angiotensin-converting enzyme inhibitors), anticholinergics, and anticonvulsants. Causality assessment classified eight reactions (80%) as probable and two (20%) as possible; none was classified as definite or doubtful. Severity assessment using the Modified Hartwig and Siegel scale classified most reactions as moderate (70%), and preventability assessment using the Schumock and Thornton criteria identified over half as probably preventable (60%).

Conclusion: Polypharmacy necessitates vigilant, proactive monitoring for oral mucosal and functional toxicity. Active interdisciplinary collaboration between a dentist, pharmacologists, and community medicine physicians can facilitate recognition and assessment of suspected oral ADRs, early mitigation of drug-induced oral morbidity, and improved patient care.

## Introduction

The WHO defines an adverse drug reaction (ADR) as a response to a medication that is noxious and unintended and that occurs at doses normally used in humans [1]. Adverse drug reactions have a negative effect on patients' quality of life in addition to placing further strain on the healthcare system, and they pose a serious threat to public health owing to increasing morbidity and mortality [2]. Polypharmacy, often arising in the management of multimorbidity and sometimes reinforced by prescribing cascades, is common in geriatric and chronic disease care [3]. The consequences of polypharmacy extend beyond the oral cavity and may include gastrointestinal disturbances, cardiovascular effects, central nervous system effects, renal or hepatic toxicity, and drug-drug interactions, depending on the medications involved [4]. In older adults, age-related physiological changes, multimorbidity, and polypharmacy may further increase susceptibility to medication-related harm [4]. While clinical guidance and medication-safety frameworks emphasize the systemic risks and medication-related harm associated with polypharmacy [4], ADRs affecting the oral cavity may be overlooked or under-recognized because of limited awareness and training among healthcare professionals [5].

Drug-induced oral manifestations are highly variable, ranging from subjective symptoms such as dysgeusia and xerostomia [6] to clinically significant complications. Xerostomia, particularly when persistent, may reduce salivary protection and increase the risk of dental caries and other oral complications [6]. More severe drug-associated manifestations include erythema multiforme [7], lichenoid drug eruptions [8], gingival overgrowth [9], and medication-related osteonecrosis of the jaw (MRONJ) [10]. These reactions significantly compromise oral health-related quality of life, often leading to secondary complications such as opportunistic infection, accelerated dental caries, and nutritional deficiency arising from impaired mastication and deglutition [6,11].

Diagnosing an oral ADR in the context of polypharmacy is a complex clinical challenge. The superimposition of age-related physiological changes, cumulative anticholinergic burden [6], and synergistic drug-drug interactions [4] complicates causality assessment [12]. This study aimed to generate preliminary pharmacovigilance signals by prospectively characterizing oral ADRs among patients receiving polypharmacy, assessing the suspected drug-ADR relationship using standardized pharmacovigilance tools, and describing their severity and preventability.

## Materials and methods

This prospective, observational, descriptive case series was conducted over six months from March to August 2026 at a tertiary care teaching hospital to capture emergent safety signals relating to oral ADRs. Ten consecutive adult patients on polypharmacy who developed new-onset oral lesions or functional oral impairment were enrolled. The evaluation was conducted through interdisciplinary collaboration. An external dental practitioner, independent of the study institution, performed the clinical oral examinations and characterized the oral manifestations. The Department of Pharmacology coordinated medication reconciliation and assessment of suspected drug-ADR causality, while the Department of Community Medicine supported patient profiling and descriptive analysis. Findings from the clinical and pharmacovigilance assessments were integrated for overall case evaluation.

Causality was assessed using the Naranjo Adverse Drug Reaction Probability Scale, a validated 10-item algorithm that scores variables such as temporal sequence, alternative etiologies, and response to dechallenge, categorizing the reaction as definite (≥9), probable (5-8), possible (1-4), or doubtful (≤0) [13]. For patients receiving multiple potentially contributory medications, causality assessment was performed with respect to the primary suspected offending drug, while concomitant medications were considered as potential confounders. 

The severity of each reaction was additionally graded using the Modified Hartwig and Siegel Severity Assessment Scale, which classifies reactions as mild (levels 1-2), moderate (levels 3-4), or severe (levels 5-7) according to the treatment, monitoring, and escalation of care required [14]. Preventability was assessed using the Schumock and Thornton criteria, which classify each reaction as definitely preventable, probably preventable, or not preventable based on factors such as the predictability of the reaction, adherence to recommended dosing and monitoring practices, and the adequacy of patient education or precautionary measures [15].

Study design

Inclusion and Exclusion Criteria

Patients were eligible if they were aged ≥18 years, had documented concurrent daily use of five or more systemic medications (including over-the-counter and complementary therapies) for a minimum of 30 days [3], and presented with a new-onset oral lesion or functional impairment temporally associated with the current medication regimen. Patients were excluded if there was an established local cause for the oral condition (e.g., ill-fitting dentures causing traumatic ulceration), a known pre-existing autoimmune vesiculobullous or mucosal disease, a history of head and neck radiotherapy (radiation-induced mucositis), or refusal to provide consent. These criteria were applied to strengthen the attribution of the oral reaction to the suspected drug rather than to a confounding cause.

Study Procedure

Each patient underwent a comprehensive clinical workup comprising demographic profiling, meticulous medication reconciliation, a detailed morphologic description of the oral lesion, and relevant laboratory or microbiological investigation where indicated. Medication reconciliation included generic drug name, dose, route, frequency, indication, duration of therapy, recent medication changes, and relevant over-the-counter or complementary medications. All suspected reactions identified during the study period were reported through the institutional pharmacovigilance mechanism, and patients who consented were enrolled using the informed consent form (ICF).

Ethical Considerations

This study was conducted in accordance with the institutional research policy of the National Institute of Medical Sciences University (NIMS), Jaipur, India. According to institutional policy, formal Institutional Ethics Committee review was not required for this non-interventional pharmacovigilance case series. Written informed consent was obtained from all participants before collection and use of clinical information and clinical photographs for research and scientific publication. Clinical photographs were obtained only when clinically appropriate and available. Patient confidentiality and anonymity were maintained throughout.

Statistical Analysis

Data were entered and analyzed using descriptive statistical methods. Continuous variables, including age and number of concurrent medications, were summarized using the mean and range. Categorical variables, including sex, oral ADR manifestations, suspected drug classes, Naranjo causality categories [13], Modified Hartwig and Siegel severity categories [14], and Schumock and Thornton preventability categories [15], were summarized as frequencies and percentages. For each case, the Naranjo score, severity category, and preventability category were assigned according to the respective predefined criteria [13]. Results are presented in tables and figures to describe the distribution and clinical characteristics of the observed oral ADRs. Given the small sample size, descriptive case-series design, and absence of a comparator or control group, no inferential statistical tests, hypothesis testing, or measures of association were performed. No imputation of missing data was required.

## Results

Ten patients experiencing oral ADRs occurring while receiving polypharmacy were evaluated over the study period. The most common presenting features were oral dryness, gingival enlargement, mucosal soreness, and a burning sensation. Hypertension and type 2 diabetes mellitus were the most frequent underlying comorbidities, and the medication burden ranged from five to eight concurrent systemic drugs. The age of the patients ranged from 55 to 80 years, with a mean of 67.8 years. They were taking five to eight medications and were evaluated via an interdisciplinary protocol. The patients in this case series were predominantly older adults with multiple comorbidities. Six of the 10 patients were male (60%), and four were female (40%). The baseline characteristics of the cohort are summarized in Table 1.

**Table 1:** Baseline characteristics of the cohort (total n = 10) ADRs: Adverse drug reactions, MRONJ: Medication-related osteonecrosis of the jaw

Characteristics		Value
No. of patients (n)	Age, mean (range), years	67.8 (55-80)
	Female, n (%)	4 (40%)
	Male, n (%)	6 (60%)
	Concurrent systemic medications, range	5-8
Comorbidities	Hypertension, n (%)	4 (40%)
	Type 2 diabetes mellitus, n (%)	5 (50%)
Oral ADRs	Xerostomia, n (%)	2 (20%)
	Gingival overgrowth, n (%)	2 (20%)
	Oral lichenoid reaction, n (%)	1 (10%)
	Oral erythema multiforme, n (%)	1 (10%)
	Pseudomembranous candidiasis, n (%)	1 (10%)
	Angioedema, n (%)	1 (10%)
	Oral mucositis/ulceration, n (%)	1 (10%)
	Suspected early MRONJ, n (%)	1 (10%)

Pattern of ADRs

The manifestations were broadly categorized into functional impairments and structural or mucosal anomalies. Drug-induced xerostomia (patients #2 and #5) and gingival overgrowth (patients #1 and #3) were the most frequently encountered ADRs, each accounting for 20% of cases. The remaining reactions (oral lichenoid reaction, erythema multiforme, pseudomembranous candidiasis, angioedema, oral mucositis, and osteonecrosis of the jaw) each occurred in a single patient.

Comprehensive patient review

The suspected offending agent, clinical manifestation, causality category, and management of each patient are presented in Table 2. The most frequently implicated agents were antihypertensives (amlodipine, captopril, and enalapril), anticonvulsants (phenytoin and carbamazepine), and drugs with prominent anticholinergic activity (amitriptyline). The patient with suspected MRONJ had a documented history of oral alendronate exposure and compatible clinical findings; alternative causes, including previous head and neck radiotherapy, were excluded.

**Table 2:** Clinical profile, causality assessment, and management of each patient MRONJ: Medication-related osteonecrosis of the jaw, ACE: Angiotensin-converting enzyme, F: Female, M: Male

Patient no.	Age/sex	Suspected offending drug(s)	Clinical oral manifestation	Naranjo score (category)	Interdisciplinary management
1	65/M	Amlodipine	Fibrotic gingival overgrowth	6 (probable)	Calcium channel blocker substituted; non-surgical periodontal scaling performed
2	72/F	Amitriptyline (primary suspected drug); furosemide as concomitant medication	Severe xerostomia	7 (probable)	Amitriptyline dose tapered; topical saliva substitutes and lubricants prescribed
3	58/M	Phenytoin	Fibrotic gingival overgrowth	8 (probable)	Neurology consultation for transition to levetiracetam; plaque control
4	61/F	Captopril	Oral lichenoid reaction	4 (possible)	Managed symptomatically with topical corticosteroid (triamcinolone acetonide 0.1%)
5	68/M	Hydrochlorothiazide (primary suspected drug); digoxin as concomitant medication	Xerostomia	5 (probable)	Systemic hydration protocol initiated; alcohol-based mouthwashes avoided
6	74/F	Carbamazepine	Oral erythema multiforme	6 (probable)	Immediate withdrawal of suspected drug; short course of systemic corticosteroid
7	80/M	Fluticasone propionate inhaler	Pseudomembranous candidiasis	7 (probable)	Topical clotrimazole prescribed; re-education on spacer use and post-inhalation rinsing
8	55/M	Enalapril	Angioedema (lip and tongue)	6 (probable)	Angiotensin-converting enzyme (ACE) inhibitor withdrawn; acute supportive management provided, with H1-antihistamine administered empirically.
9	67/M	Methotrexate	Oral mucositis/ulceration	7 (probable)	Rheumatology-coordinated folic acid supplementation; symptomatic relief with benzydamine
10	78/F	Alendronate (oral)	Suspected early MRONJ	4 (possible)	Conservative debridement and 0.12% chlorhexidine gluconate irrigation; drug holiday discussed with endocrinologist

Causality assessment

Application of the Naranjo scale indicated a strong temporal and clinical correlation. Eight reactions (80%) fell within the probable category, supporting a probable association with the suspected drug despite the presence of concomitant medications, while the remaining two (20%) were classified as possible (Figure 1). Management generally involved an interdisciplinary approach combining pharmacological modification (dose adjustment, substitution, or definitive withdrawal) with targeted dental and mucosal therapy.

**Figure 1:**
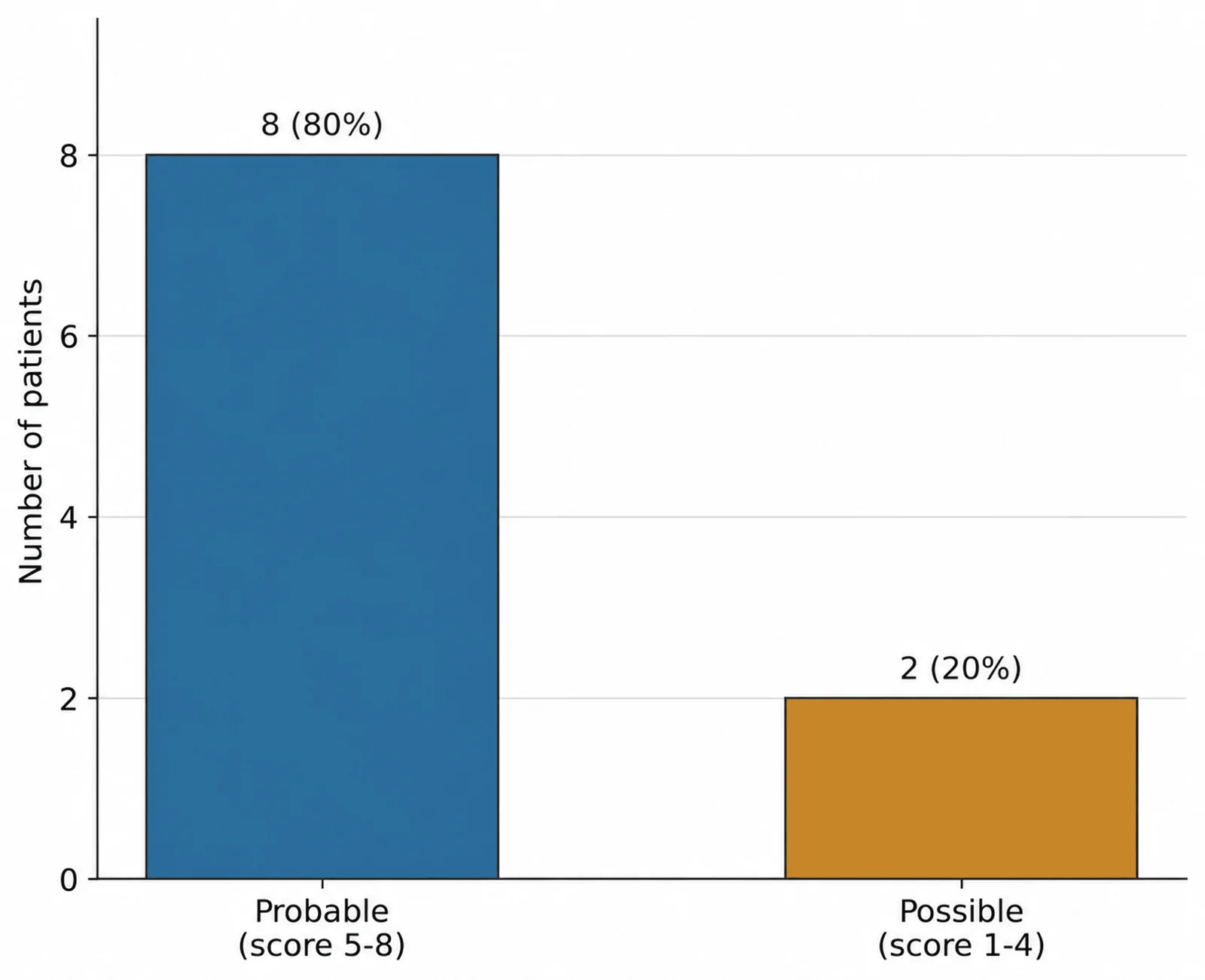
Distribution of patients by Naranjo causality category Eight patients (80%) had reactions classified as probable and two (20%) as possible; none was classified as definite or doubtful.

Severity and preventability assessment

Applying the Modified Hartwig and Siegel Severity Assessment Scale, seven reactions (70%) were classified as moderate (levels 3-4), reflecting the need for a change in treatment, specialist referral, or targeted dental intervention, while three (30%) were classified as mild (level 2); none was classified as severe (levels 5-7). Preventability assessment using the Schumock and Thornton criteria classified one reaction (10%) as definitely preventable, six (60%) as probably preventable, and three (30%) as not preventable (Table 3). Immune-mediated reactions such as the oral lichenoid reaction, erythema multiforme, and angioedema were considered not preventable given their idiosyncratic, hypersensitivity-based mechanisms, whereas dose- and technique-related reactions such as gingival overgrowth, xerostomia, oral mucositis, and pseudomembranous candidiasis were considered probably or definitely preventable with closer monitoring or patient education.

**Table 3:** Severity and preventability assessment of oral ADRs Severity categories: Mild (levels 1-2), moderate (levels 3-4), severe (levels 5-7); ADRs: Adverse drug reactions, MRONJ: Medication-related osteonecrosis of the jaw

Patient no.	Clinical oral manifestation	Modified Hartwig and Siegel severity (level and category)	Schumock and Thornton preventability assessment
1	Fibrotic gingival overgrowth	3 (moderate)	Probably preventable
2	Severe xerostomia	3 (moderate)	Probably preventable
3	Fibrotic gingival overgrowth	4a (moderate)	Probably preventable
4	Oral lichenoid reaction	2 (mild)	Not preventable
5	Xerostomia	2 (mild)	Probably preventable
6	Oral erythema multiforme	4a (moderate)	Not preventable
7	Pseudomembranous candidiasis	2 (mild)	Definitely preventable
8	Angioedema (lip and tongue)	4a (moderate)	Not preventable
9	Oral mucositis/ulceration	3 (moderate)	Probably preventable
10	Suspected early MRONJ	4b (moderate)	Probably preventable

## Discussion

This prospective case series illustrates the spectrum of oral ADRs observed among patients receiving polypharmacy. By evaluating these cases through a collaborative, interdisciplinary lens, the study illustrates how distinct pharmacological mechanisms translate into clinically significant oral pathology, a domain often underrepresented in standard adverse-event reporting systems [5].

Functional impairment (the cumulative anticholinergic burden)

Xerostomia emerged as a prominent complication, particularly in patients receiving combined diuretic and psychotropic therapy (e.g., furosemide and amitriptyline). Xerostomia may result from anticholinergic effects on salivary secretion, with other factors such as concomitant diuretic therapy, reduced fluid intake, and age-related changes potentially contributing to symptoms [6]. This reduction in salivary flow is not merely a subjective nuisance; it fundamentally alters the oral microenvironment, depleting the mucosa of protective salivary constituents and buffering capacity and thereby predisposing the patient to accelerated cervical caries and opportunistic infection [6]. The latter is exemplified by the pseudomembranous candidiasis observed in a patient using an inhaled corticosteroid, in whom local immunosuppression and reduced mucosal clearance favored *Candida* overgrowth; reinforcement of spacer use and post-inhalation rinsing is central to prevention [16].

Structural and mucosal modifications

Drug-induced gingival overgrowth was classically observed with amlodipine and phenytoin. Although the pathogenesis is multifactorial, these agents alter intracellular calcium handling in gingival fibroblasts, producing a localized imbalance between synthesis and degradation of the extracellular matrix, with accumulation of collagen and dysregulation of matrix metalloproteinases [9]. Successful management required a coordinated effort, namely medical substitution of the offending agent alongside rigorous periodontal phase I therapy, emphasizing that pharmacological alteration alone is often insufficient without local plaque control [9].

Immune-mediated mucosal reactions, such as the lichenoid reaction associated with captopril [8] and the potentially life-threatening erythema multiforme triggered by carbamazepine [7], highlight the need to recognize the oral mucosa as a primary site for systemic hypersensitivity. The angioedema of the lip and tongue seen with enalapril is consistent with the predominantly bradykinin-mediated mechanism of ACE inhibitor-associated angioedema. The ACE inhibitor was promptly withdrawn, while acute supportive management was provided; conventional antihistamines may be administered empirically but are not reliably effective in bradykinin-mediated angioedema [17]. For methotrexate-induced mucositis, the mechanism relates to folate antagonism and inhibition of DNA synthesis in the rapidly dividing basal cells of the oral epithelium, a toxicity effectively mitigated through targeted folic acid rescue [18]. Finally, the suspected early medication-related osteonecrosis of the jaw in a patient receiving oral alendronate underscores the value of conservative debridement, antiseptic irrigation, and multidisciplinary liaison with the prescribing physician rather than aggressive surgery in the first instance [10].

The role of active pharmacovigilance and education

Pinpointing a single causal agent in a patient consuming up to eight medications is inherently challenging. Synergistic interactions frequently amplify toxicity; for instance, a diuretic exacerbating the mucosal dryness caused by an antidepressant [12]. In this setting, the Naranjo scale provided a standardized framework for structuring clinical causality assessment; however, attribution remained challenging because of concomitant medications and potential alternative causes [13]. Complementing causality assessment, the Modified Hartwig and Siegel severity scale and the Schumock and Thornton preventability criteria provided additional structure for characterizing the clinical impact and avoidability of these reactions, consistent with their established use in other pharmacovigilance case series [14,15]. Although the study is limited by its small sample size, single-center design, and absence of a control cohort, it provides useful preliminary observations consistent with previous reports of ADRs among older adults receiving polypharmacy [11]. Previous pharmacovigilance work has also documented clinically important adverse drug reactions in a different clinical setting, where systematic causality assessment using the Naranjo scale has similarly identified probable drug-induced reactions ranging from valproate-associated hyperammonemia [19] to Stevens-Johnson syndrome caused by antimicrobial and anti-epileptic agents [2], underscoring the broader value of structured, center-based surveillance across organ systems and drug classes. More importantly, the present findings highlight a broader systemic need: the integration of localized pharmacovigilance training into continuing health-profession education so that clinical faculty and students learn to inspect the oral cavity during routine medication review, an inexpensive step that could substantially reduce drug-induced oral morbidity [5].

Limitations

The small sample size and single-center design limit the generalizability of the findings and preclude estimation of the incidence or prevalence of individual oral ADRs. The absence of a comparator group prevents assessment of the relative contribution of polypharmacy to these manifestations. Causal attribution was also challenging because several patients were receiving multiple concomitant medications, and some oral conditions may have multifactorial etiologies. Intentional rechallenge was not performed for ethical reasons. The findings should therefore be interpreted as descriptive clinical observations and hypothesis-generating pharmacovigilance signals rather than estimates of population-level risk.

## Conclusions

Oral ADRs are clinically important manifestations that may be overlooked during routine medication review, particularly among patients receiving multiple medications. In this case series, xerostomia and gingival overgrowth were among the most frequently observed manifestations, while antihypertensives, anticonvulsants, and drugs with prominent anticholinergic activity were frequently suspected medication classes. Structured assessment of causality, severity, and preventability, together with interdisciplinary collaboration between dental practitioners, pharmacologists, and prescribing physicians, supported the recognition and management of suspected oral ADRs. Routine oral assessment during medication review may help improve the recognition and appropriate management of oral ADRs. Given the small sample size and exploratory case-series design, these findings should be considered preliminary pharmacovigilance observations rather than estimates of ADR frequency or definitive evidence of causality. Larger prospective studies are warranted to further evaluate these observations.
